# An Approach to Frequency Selectivity in an Urban Environment by Means of Multi-Path Acoustic Channel Analysis

**DOI:** 10.3390/s19122793

**Published:** 2019-06-21

**Authors:** Pau Bergadà, Rosa Ma Alsina-Pagès

**Affiliations:** 1 Grup de recerca en Tecnologies Mèdia (GTM), La Salle, Universitat Ramon Llull, c/Quatre Camins, 30, 08022 Barcelona, Spain; pbergadac@gmail.com; 2 Wavecontrol, c/Pallars, 65-71, 08018 Barcelona, Spain

**Keywords:** noise, propagation model, frequency selectivity, acoustic channel, wideband, auralization, smartcity, impulse response, coherence bandwidth, wireless acoustic sensor network

## Abstract

The improvement of quality of life in the framework of the smart city paradigm cannot be limited to a set of objective measures carried out over several critical parameters (e.g., noise or air pollution). Noise disturbances depend not only on the equivalent level *L_Aeq_* measured, but also on the spectral distribution of the sounds perceived by people. Propagation modelling to conduct auralization can be done either with geometrical acoustics or with wave-based methods, given the fact that urban environments are acoustically complex scenarios. In this work, we present a first analysis of the acoustic spectral distribution of street noise, based on the frequency selectivity of the urban outdoor channel and its corresponding coherence bandwidth. The analysis was conducted in the framework of the data collected in the Milan pilotWASN of the DYNAMAP LIFE project, with the use of three simulated acoustic impulse responses. The results show the clear influence of the evaluated coherence bandwidth of each of the simulated channels over real-life acoustic samples, which leads us to the conclusion that all raw acoustic samples have to be considered as wide-band. The results also depict a dependence of accumulated energy at the receiver with the coherence bandwidth of the channel. We conclude that, the higher the delay spread of the channel, the narrower the coherence bandwidth and the higher the distortion suffered by acoustic signals. Moreover, the accumulated energy of the received signal along the frequency axis tends to differ from the accumulated energy of the transmitted signal when facing narrow coherence bandwidth channels; whereas the accumulated energy along the time axis diverges from the accumulated transmitted energy when facing wide coherence bandwidth channels.

## 1. Introduction

As a result of population growth and the consequent expansion of transportation systems, including highways, railways, and airways, environmental noise pollution has been increasing. Noise pollution continues to constitute a major environmental health problem in Europe [1,2]. Among the health effects, annoyance is one of the principal environmental noise [3] issues; however, it is not merely an annoyance, as several works have detected health problems, such as sleep disorders [4], learning impairment [5], and heart diseases [6]. Thus, noise impact is one of the main environmental health concerns [7], and the harmful effects it causes on social and economic aspects have been proved [8].

The European Union reacted to this alarming increase of environmental noise pollution, especially in densely populated cities, with the Environmental Noise Directive 2002/49/EC (END) [9]. In accordance with the END, the CNOSSOS-EU methodological framework aims to improve the consistency and comparability of noise assessment results across the EU Member States [10] for its application. The main pillars of the END are the following: (i) Determining the noise exposure; (ii) updating information related to the noise available to citizens; and (iii) preventing and reducing environmental noise, where necessary.

Recent studies have showed that the effects of noise on people do not only depend on the level of noise, but also on the type of sound. In fact, in 2018, the WHO incorporated noises, such as leisure noise and wind turbine noise [11]. To accomplish the goal of measuring each type of noise source, the Anomalous Noise Event Detector (ANED) [12] was designed by this team to rule out non-road traffic noise (RTN) events from road traffic noise measurements. The ANED is an algorithm based on the spectral distribution of the different types of RTN and anomalous noise events (ANE) in order to properly identify them and, in this study, it is proven that the sound propagation and its impact on spectral behavior may change its performance [13]. Furthermore, by changing the temporal and spectral distribution of the signal, human perception may also change with respect to a reference noise measured in outdoor environmental conditions [14]. Our team has begun work on the evaluation of the perception of certain types of sound in outdoor conditions [15,16], with promising results that are still under study in the urban environment of Rome.

In an urban environment, a detailed study and simulated reproduction of the propagation of a sound—as it will be perceived by people—is a key factor in the evaluation and prediction of how people will react to the noise [3]; this approximation is called auralization [17]; virtual reality has even been used to reproduce the audio-visual environment [18]. Propagation modelling, with the final goal of auralization, has been proposed in the literature from two points of view: (i) Geometrical acoustics, and (ii) wave-based methods [19]. The analysis detailed in this work is based on the wide-band channel sounding principles which are widely used in communications [20], which demonstrate the usefulness of studying channel fading [21,22]. The techniques of channel estimation, taking into account multi-path propagation and its subsequent coherence bandwidth, have been found to be useful in acoustic propagation environments, mainly in underwater channels [23,24]. Several studies have been conducted in this area, with analysis of the scattering function, multi-path intensity profile, the coherence of an underwater acoustic channel [25], and analysing the impact of the coherence bandwidth on the transmission of pressure waves in image transmission [26].

The authors have made the assumption that the acoustic recordings are wide-band, given that the influence of the channel is in-band frequency selective [19]. The final goal of this preliminary study is to accurately determine whether the frequency selectivity of the channel changes the spectral distribution of several recorded acoustic raw signals [27]. The impulse response of the outdoor urban environment corresponds to the simulation of three different multi-path acoustic channels. We accurately describe each impulse response of the channel and its effects on the spectral distribution of real-life acoustic data collected in the framework of the DYNAMAP pilot project carried out in Milan, focusing on the coherence bandwidth. This work intends to be a first step in the analysis of the frequency selectivity applied to non-traffic-related noise, in order to evaluate the effect of the channel [28] on the spectrum-temporal vision of several real-operation raw signals collected in the Milan pilot of project DYNAMAP [29,30], with the final goal of its impact on auralization.

This paper is structured as follows. Section 2 details the methodology used to conduct this analysis. Section 3 details the conditions under which the acoustic raw data were collected in a real-operation environment. Section 4 details the mathematical models used to simulate the propagation impairments by means of pseudo-noise sequences. Section 5 details the results of the propagation of the simulated channels using real-life data and, finally, Section 6 details the conclusions and future work.

## 2. Overview of Methodology

The study we present in this paper uses the basis of the application of propagation modelling methods in an outdoor environment with the concept of wide-band sounding using pseudo-noise (PN) sequences, mostly used in channel soundings in communications. The acoustic communication systems are considered to be wide-band, since the channel coherence bandwidth is similar, or even narrower, than the acoustic signal bandwidth.

### 2.1. Outdoor Acoustic Propagation Modelling Basics

Propagationmodellingwith auralization purposes can be done bothwith geometrical acoustics and wave-based methods [19]. Urban sound propagation modelling should take into account the reflections coming from facades and other reflection surfaces, which might be specular or diffuse [31,32,33]. Each element has its own frequency-dependent reflection properties, which modifies the phase and the amplitude of the acoustic wave, accordingly.

Furthermore, modelling of the diffraction corresponding to the acoustic wave should also be included; in an urban environment, they often occur with multiple building edges, roofs, and corners [34]. Additionally, meteorological conditions will affect at least the long-range sound propagation; wind and turbulence also play a role in this [35]. This brief review of the most relevant elements for modelling the acoustic channel in a city demonstrates its complexity.

This work does not intend to deal with this in depth but, rather, the influence of the most basic parameters of acoustic propagation (e.g., attenuation, reflection, and influence of the medium of propagation) from a real network.

### 2.2. Wide-Band Channel Sounding with PN-Sequences

Any wide-band sounding focuses on the analysis of time-frequency dispersive features of the channel under study. Time dispersion [20] is the time-spread suffered by any wave when propagated in a medium. This corresponds to the interval of delay that causes that the auto-correlation function of the channel impulse response to differ from zero in the receiver. Frequency dispersion [20] describes the channel variation speed; the Doppler spread is the frequency range of the channel impulse response auto-correlation function that differs from zero [36].

#### 2.2.1. PN-Sequence Wide-Band Analysis Proposal

**Figure 1:**
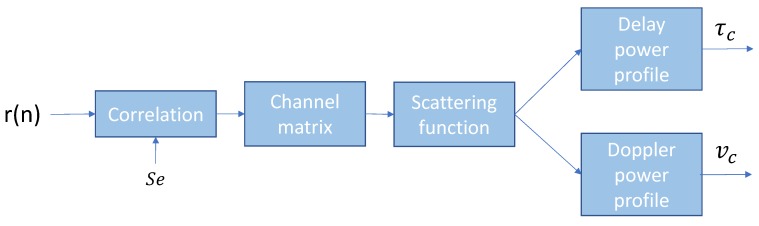
Diagram of the computation process for wide-band channel sounding.

There are several methods to conduct wide-band sounding and channel analysis. In this work, we have used the transmission of PN waveforms [37] with good cyclic cross-correlation characteristics, such as m-sequences [20]. The computation process for the wide-band sounding is detailed in Figure 1.

The signal *r*[*n*] in the receiver is correlated with the original PN sequence *S_e_* shaped by a raised cosine filter with a determined roll-off factor, which is evaluated depending on the application. The correlation function is calculated as:

(1) $$\phi_{r\left[n\right],S_{e}}\left[n\right]=\sum_{k=0}^{N_{e}-1}r\left[n+k\right]S_{e}\left[k\right],$$

where *N_e_* is the length of the PN sequence and *S_e_* is the sequence filtered with a raised cosine filter. Then, the channel impulse response *h*[*n*, *τ*] can be written as:

(2) $$h\left[n,\tau \right]=\phi_{r,S_{e}}\left[nlN_{c}+\tau \right],$$

where *τ* is the variable corresponding to the delay, *l* is the number of chips [20] (i.e., a pulse of a PN sequence), and $N_{c}$ is the number of samples per chip. From Equation (2), we can evaluate the scattering function, which leads us to the multi-path and Doppler spreads caused by the variant channel. The scattering function $R_{s}\left[\tau ,v\right]$ is calculated as the Fourier transform of the channel impulse response [20]:

(3) $$R_{h}\left[\xi ,\tau \right]=\sum_{\xi }h^{*}\left[n,\tau \right]h\left[n+\xi ,\tau \right],$$

(4) $$R_{s}\left[\tau ,v\right]=\sum_{\xi }R_{h}\left[\xi ,\tau \right]e^{-j2\pi \xi v}.$$

Both the multi-path spread ($\tau_{c}$) and the Doppler spread ($v_{c}$) can be computed from the scattering function $R_{s}\left[\tau ,v\right]$ using a certain observation window, which is wider or narrower depending on the channel variations and the application under study.

#### 2.2.2. Underwater Acoustic Channel Sounding

The acoustic communication channel may have a sparse impulse response, where physical paths act as time-varying low-pass filters and where movement introduces both Doppler spread and shift. One of the applications of PN-sequence wide-band sounding is the field of underwater acoustic channel communications. Underwater acoustic channels are usually catalogued as one of the most hostile communications systems [38]. As the bandwidth is extremely limited, an acoustic system may operate in a frequency range between 10 and 15 kHz; although the total bandwidth is low (around 5 kHz), the system is considered wide-band as its bandwidth is not negligible in terms of coherence bandwidth.

Several studies, in which scientists used PN-sequence based systems to conduct sounding in a wide-band acoustic communications underwater channel can be found in literature. In [39], the authors described an underwater sensor network, which they took advantage of to perform acoustic tomography. In [40], the authors described Hermes, an asymmetrical point-to-point underwater acoustic modem designed for short-range operations, and explored the possibility of its possible evolution into a multiple-input-multiple-output (MIMO) device. In [41], the authors conducted a survey in northern Europe, covering the continental shelf, Norwegian fjords, a sheltered bay, a channel, and the Baltic Sea. The sounding measurements were performed in various frequency bands between 2–32 kHz, in order to define a typical acoustic communications channel.

## 3. Real-Operation Acoustic Data Recordings in the DYNAMAP Project

In this section, we describe the real-operation conditions used to record the acoustic raw data used for the analysis. First, we describe the recording campaign in Milan by means of a WASN. Then, we detail which of the sensors of the entire WASN were chosen to analyse their data and, finally, we describe the types of noise event used for this test and our reasons for their selection.

### 3.1. The DYNAMAP Project

The DYNAMAP LIFE project proposed the implementation of a dynamic noise mapping system [29], able to determine the acoustic impact of road infrastructures in real-time, following the European Noise Directive 2002/49/EC. A Multi-Sensor Network collects the noise level measurements in two pilot areas: In the city of Milan, and on the A90 motorway around Rome.

Each of the sensor nodes has to accomplish a set of basic specifications [42] defined to satisfy DYNAMAP requirements for each monitoring station, which are the following: (i) A 40–100 dB(A) broadband linearity range; (ii) a 35–115 dB working range with acceptable Total Harmonic Distortion (THD); (iii) a narrow-band floor noise level; and (iv) a sampling rate of 48 kHz. The project also requires the possibility of audio recording, as well as VPN and GPRS/3G/Wi-Fi connections. The precision of the sensors is a key issue for system reliability [43].

**Figure 2:**
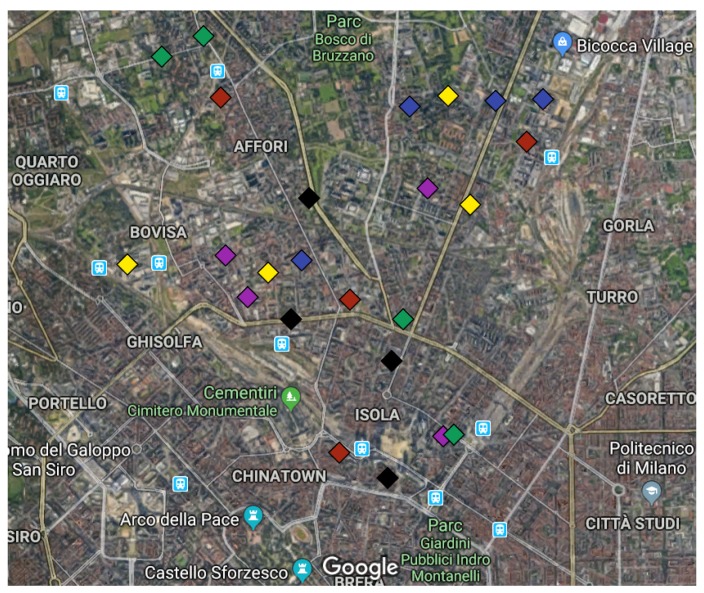
Map of the sensor locations in Milan. Different colors correspond to a catalog of six groups of streets [46].

In the case of the city of Milan, the deployment of the network is shown in Figure 2, where a team of acoustic experts chose up to 24 low-cost sensor locations to collect the data to generate noise maps. All of these locations are a key issue for the study of the features of the recorded signal and, therefore, for the performance of the ANED [12]. For more details about the location of the sensors, the reader should refer to [44,45].

Although the final goal of the DYNAMAP project is to dynamically update the noise maps in a GIS-based platform [29,47], preliminary work has been done in the sensors to evaluate the features of the raw acoustic signals collected by the network; this work enabled the recollection of up to 100 h of outdoor urban acoustic raw data in the Milan environment. 

### 3.2. Description of the Recording Campaign

**Figure 3:**
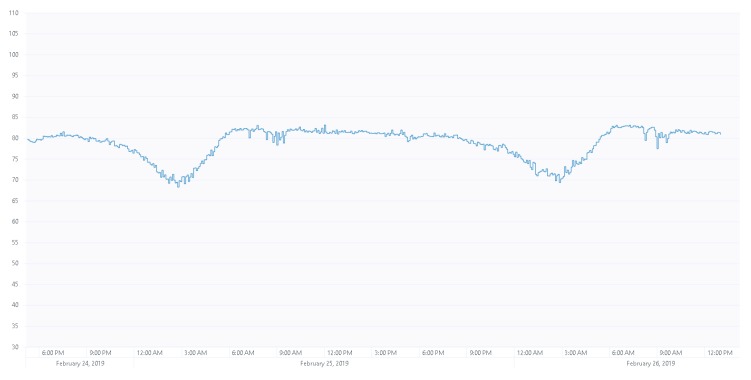
Example of two-day $L_{Aeq300}$ curve of sensor hb148. The vertical axis is the measured $L_{Aeq300}$, in dB.

Table 1 lists the sensor nodes of the WASN, indicating their identifiers, street location, and GPS coordinates within district nine of Milan, in which they were installed. A recording campaign considering both weekends and weekdays (recording 20 min each hour every day) was performed through the multi-sensor network in real-operation conditions. The final selection of files to be labelled allowed our team to collect more than 100 h of raw urban acoustic data. Previous studies [48] showed that sampling two different days could accomplish the requirements of diversity of the urban activity during weekend days and weekdays; the different traffic flow attains both RTN and also the anomalous noise events found in an urban environment, showing the relevant differences. These previous studies took into account the experience of data set designs for other acoustic event detection projects, such as those in [8,49]. Furthermore, the recordings were exhaustive, considering the entire day period of each day, for two reasons: (i) The road traffic noise profile changes between day and night, as can be observed in the two-day equivalent noise level curve in Figure 3—noise profile curves attain a certain 24 h regularity, and this property has been taken into account in the recordings; and (ii) because the types of events occurring are substantially different between day and night, and recording with a periodic sample widens the possibility of collecting more types of noise.

To that effect, recordings were planned for the 24-node network during the first 20 min of each hour, for each hour of the day, during two selected days: A weekday (Tuesday, 28th of November 2017) and a weekend day (Sunday, 3rd of December 2017), in order to maximize the diversity of the recorded ANEs and with a schedule intended to be exhaustive. A total of 1116 recordings of 20 min each were gathered during this recording campaign, producing an audio database of 372 h, from which our research group managed to manually listen and label up to 100 h.

### 3.3. Acoustic Environment of the Nodes of the WASN

There are currently 24 low-cost high-capacity sensors deployed in a WASN in district nine in Milan, as described in Table 1. Nevertheless, in the framework of the DYNAMAP project, the acoustic environment of the sensors has been studied [45] and, together with the description of several roads conducted by the colleagues of Universitá degli Studi di Milano Bicocca [46], we reached the conclusion that not all the acoustic data collected in all the sensors was suitable to conduct this kind of analysis. Under ideal conditions, the noise events under test should be recorded in an free-field environment; an unfeasible requirement, not only for the DYNAMAP project, but also for any other project that intends to collect urban noise events.

**Figure 4:**
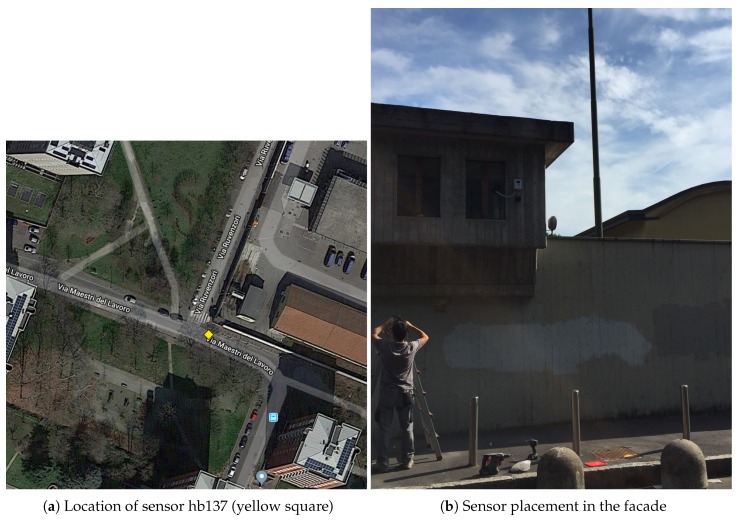
Sensor hb137 location and picture.

**Figure 5:**
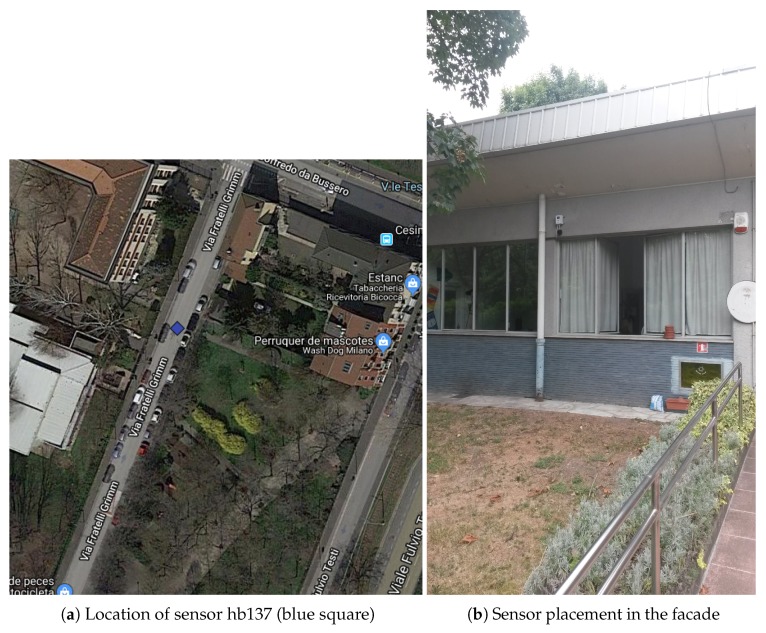
Sensor hb145 location and picture.

An analysis was conducted with the aim of determining which sensors could capture noise events in a similar way to this ideal free-field environment. The first item to consider was the placement of sensors in narrow streets with buildings, due to the acoustic effect over noise events, which substantially changes the frequency distribution of the recorded data. We finally discovered that two of the sensors (hb137 and hb145) were placed in a facade, but surrounded by parks. Sensor hb137 was located in a quiet area, surrounded by two parks with trees. Furthermore, the closest building to this sensor was more than 55 meters away (see Figure 4). Sensor hb145 was also in a narrow street, but was surrounded by a park; both of them were placed in public buildings, one of them being a school. The closest wall to the sensor was around 35 m away (see Figure 5). Nevertheless, in both locations, the sensors were placed on the façade, so the *façade effect* will appear for both recordings; in future work, the authors will consider working on its mitigation [50].

**Figure 6:**
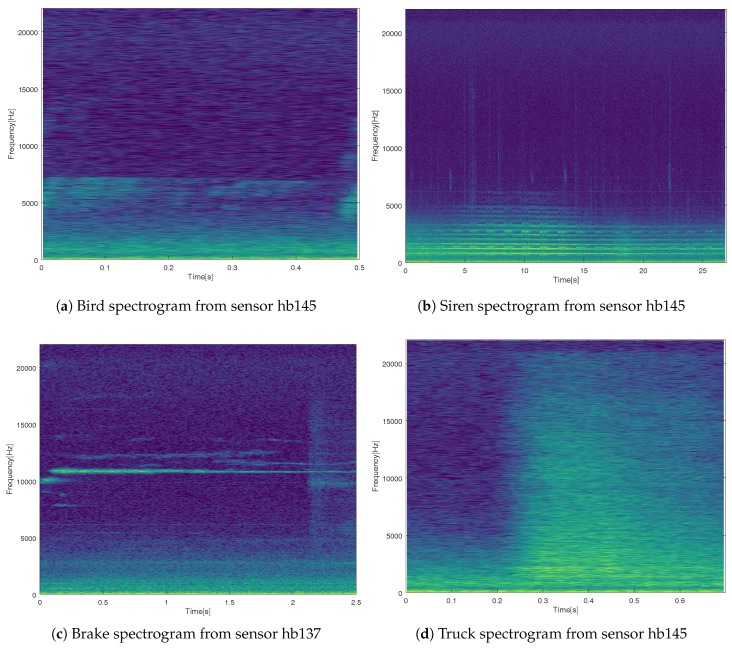
Examples of anomalous noise events recorded by sensors hb137 and hb145.

Once the sensors had been chosen, an analysis of the recorded anomalous noise events was conducted. Dozens of types of anomalous noise events were found to be recorded by those two sensors, such as: Birds (609 occurrences), brake (62), truck (20), siren (15), door (131), people (169), trolley (10), dog (134), aeroplane (28), chain (21), step (45), bell (11), bike (16), glass (3), bus door (8), saw (8), and tram (16). In Figure 6, four examples can be found of anomalous noise events recorded in sensor hb137 and hb145, showing that different ANEs present very variate frequency distributions.

The analysis of the available samples of anomalous noise events was focused on the spectral diversity and minimum length of the events. The events used for this analysis, including those from both sensors (hb137 and hb145), had an overall length of 4865 s. The maximum length of the processed ANEs was 191 s and the minimum length was 0.1 s. Another of the pursued goals was to study several types of spectral distribution, both wide-band and narrow-band.

In Figure 6, the bird spectrogram in Figure 6a presents a short audio of 0.5 s and a distribution of frequencies between 5 kHz and 7.5 kHz. The siren spectrogram in Figure 6b shows a good signal-to-noise (SNR) ratio in the raw audio, which clearly presents a Doppler effect. Even more, it was a long event, with a duration of around 25 s. In Figure 6c, a good signal-to-noise ratio brake is plotted, with a clear component around 11,000 Hz. This event was around 2.5 sec length. In Figure 6d, we can observe a wide-band signal, whose spectrum includes frequencies up to around 20 kHz. Low frequencies, between 0 Hz and 1 kHz, are occupied in all the audio pieces by road traffic noise, which presents energy mainly in those frequencies.

## 4. Channel Model Design

In this section, we define three urban channel models, computed as a linear combination of the input source and attenuated delayed paths. The three channel models are static (i.e., neither the transmitter nor the receiver changed their position while the sound signal was propagating) and, hence, no signal frequency spread and Doppler shift are expected. We defined three channel models which represent three different urban scenarios, with different number of paths and delay. The aim is to transmit the sound signals through each of the channel models and study the changes of time and frequency response.

Prior to applying the recorded sound signals to the three channel models, we characterised the channel in terms of time-spread by means of a PN sequence with good auto-correlation and cross-correlation properties [37]. The goal was to be able to detect each of the paths of the channel impulse response with a wide enough bandwidth to check whether the input sound signal had suffered any impairment throughout its whole bandwidth.

### 4.1. Outdoor Propagation Models

In this section, we describe the main characteristics of the outdoor propagation model used in this work. We consider a sound signal radiating as a spherical isotropic wave-front [51] through an homogeneous medium; that is, a medium with constant sound speed (*c*_0_). To define the sound velocity as *c*_0_ = 343.23 ≈ 343 m/s, we assume the following conditions:

- Atmospheric density and pressure are assumed constant.
- The density of the air at ground level is 1.205 kg/m^3^, the temperature is 20°, and the atmospheric pressure is 1 atm.
- The relative humidity is 70%.

In such a case, the acoustic free-field intensity of the radiation decreases with the inverse square of the distance and might travel to a receiver along a direct path and a number of reflected paths. Therefore, we can describe the acoustic pressure (*p*(*r*)) at a Euclidean distance *r* from the emitter in the following form [52]:

(5) $$p\left(r\right)=\left[\frac{A(r_{0})}{r_{0}}\right]e^{jkr_{0}}+\sum_{i=1}^{i=N}Q\left[\frac{A(r_{i})}{r_{i}}\right]e^{jkr_{i}},$$

where $r_{0}$ is the distance travelled by the direct path; $r_{i}$ the distance travelled by each of the remaining *N* paths, which may be reflected by the ground, surrounding buildings, trees, and so on; A(r) is the atmospheric attenuation; *Q* is the reflection coefficient; and $k=w/c_{0}$ is the wave number.

The atmosphere dissipates sound energy through two major mechanisms—viscous losses and relaxational processes—which have been extensively studied in the ANSI Standard S1-26:1995 [53]. The main mechanism of absorption is proportional to the square of the frequency. The relaxational processes also depend on the relaxation frequency of nitrogen and oxygen. Given the above atmospheric conditions, we can compute the attenuation suffered by an acoustic signal due to atmospheric absorption as:

(6) $$A\left(r\right)=\frac{\alpha r}{100}\;\left[\mathrm{dB}\right],$$

where *r* (in meters) is the distance between emitter and receiver and α is the absorption coefficient (in dB/100 m). Given the above meteorological conditions of temperature and relative humidity, we can take the absorption coefficient equal to 0.54 dB/100m at 1000 Hz and 10.96 dB/100m at 10,000 Hz.

The reflection coefficient is defined for spherical waves reflecting from complex plane boundary and can be approximated as:

(7) $$Q=R_{p}\left(\theta \right)+B\left(1-R_{p}\left(\theta \right)\right)F\left(w\right),$$

where $R_{p}\left(\theta \right)$ is the plane-wave reflection coefficient, θ is the glancing angle, *B* is a correction term, and F(w) is the boundary loss function defined by means of the numerical distance *w* [53]. If we assume a locally reacting ground and set B=1, then:

(8) $$R_{p}\left(\theta \right)=\frac{sin(\theta )-1/Z}{sin(\theta )+1/Z}\;,$$

where *Z* is the normalised acoustic impedance of the ground. We note that, for low glancing angles (*θ*), $R_{p}\to -1$ and, for high frequencies, F(w) diminishes and, consequently, $Q=R_{p}$.

### 4.2. Impulse Response for the Defined Acoustic Channels

We define three different urban channel models, which are linear combinations of sets of paths received at a sensor, each with an acoustic pressure and phase shift as described by Equation (9):

(9) $$y\left(n\right)=\sum_{i=1}^{N}x\left(n-\tau_{i}\right)a_{i}e^{j\tau_{i}2\pi w},$$

where y(n) is the received signal at the sensor, x(n) is the transmitted signal through each path whose amplitude (i.e., $a_{i}$) is computed as explained in Equation (5), N is the number of paths of each model, $\tau_{i}$ is the delay suffered by each path, and $e^{j\tau_{i}2*\pi w}$ is the phase delay of each path, which is proportional to the delay and frequency.

In order to study the effect of time-dispersive channels on acoustic signals, we propose three different scenarios. We first define a simple channel (model A) with just two paths, which can describe a wide street with a direct path and a reflection path on the ground. Models B and C describe two scenarios with more dense scatterers than model A: While model B depicts a scenario with a high delay spread, which may be distinctive of a wide street, model C represents a highly scattered scenario with half the delay spread of model B, which may be characteristic of a narrower street. Table 2 shows the length and delay of each path per model (where the propagation velocity was taken as 343 m/s).

**Figure 7:**
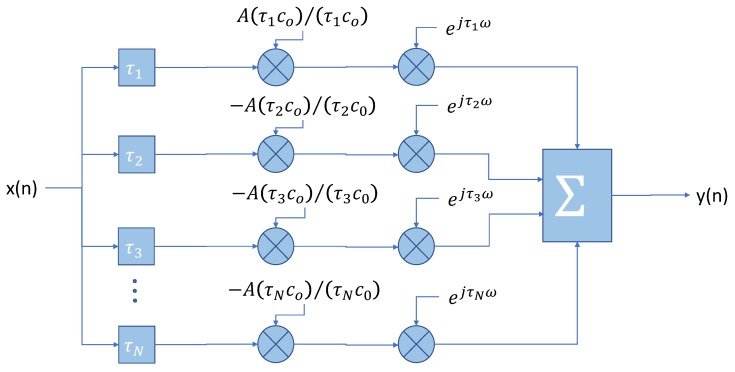
Block diagram for the N-path channel.

In Figure 7, we show a block diagram of the synthesis of the channel impulse response of a *N*-path time-dispersive channel, as described by Equation (9). The input signal x(n) is first delayed a number of samples proportional to the delay, $\tau_{i}$, suffered by each path. It is then attenuated with an inverse proportion to the distance travelled and, finally, the phase is shifted proportionally to the distance travelled.

**Figure 8:**
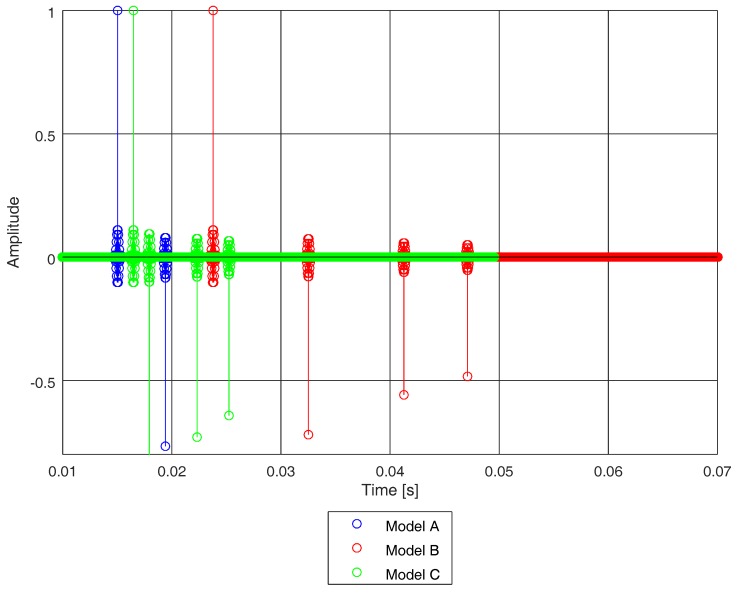
Channel ideal impulse response of models A, B, and C.

Figure 8 depicts a snapshot of the ideal impulse response of each of the three models used in this study. Each impulse response is normalised to the first path, which is the direct path and that with highest intensity at the receiver. The remaining paths are reflected paths showing negative amplitude, which denotes a value of phase between π and 2π. Model A shows two taps, whereas models B and C show four taps. The taps of model B spread in time, over a much longer period than the taps of model A and C. Therefore, it is worth noting that model B will spread the energy of the propagated signal throughout a higher time-span than models A and C, which have closer paths.

### 4.3. PN Sequence Channel Estimation

**Figure 9:**
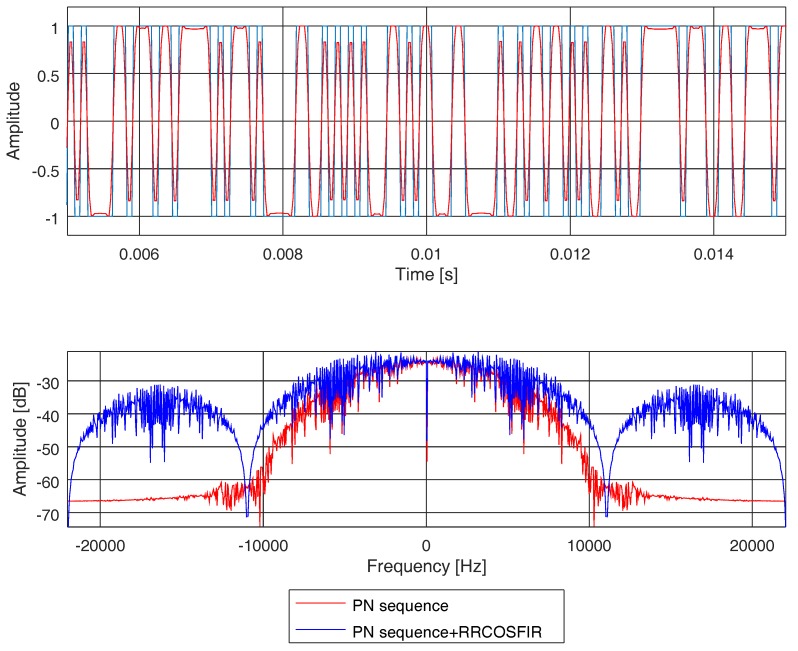
PN sequence time response and frequency response. The sequence without filtering is shown in blue, and after filtering (with a root raised cosine) is shown in red.

The goal of this section is to present a method to estimate the channel impulse response and frequency response of a time-dispersive and static channel (no movement in either transmitter or receiver is expected). In order to characterise the effects of this type of channel on acoustic signals, we use a PN sequence of type M, which has good cyclic cross-correlation properties [37]. The sequence is sampled at *F_s_* = 44.10 kHz, with 1023 chips length and four samples per chip. Then, the chip period is *T_c_* = 4/*F_s_* and, hence, the detection bandwidth equals 1/*T_c_* and the delay resolution equals *T_c_*. The PN sequence is also low-pass filtered with a Finite Impulse Response (FIR) root raised cosine filter (RRCOSFIR) with a roll-off factor equal to 0.9. The aim of this low-pass filtering is to limit the bandwidth of the PN sequence to 10 kHz, which is the most common band for audible signals. In Figure 9, we show the time domain (upper figure) and frequency domain (lower figure) of the PN sequence (in blue), as well as the same PN sequence after filtering (in red). It is worth noting that the time response displays a pseduo-random nature and that the bandwidth is limited after being filtered.

The analysis of the channel by means of a PN sequence is a key factor to characterise the time dispersion and, hence, to evaluate whether the signal arrived with any multi-path component at the receiver. First, the received signal x[n] is correlated with the original PN sequence *S*. The correlation function is computed as explained in Equation (1).

**Figure 10:**
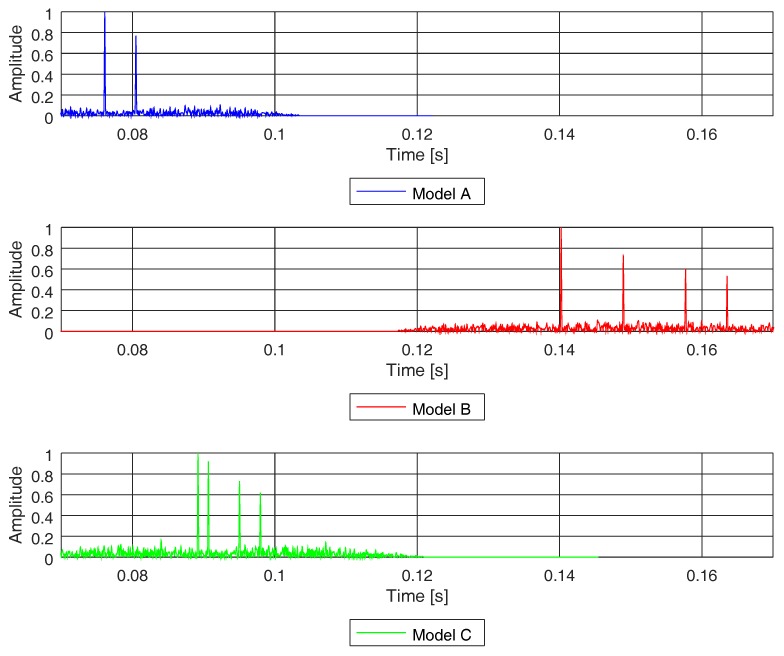
Channel impulse responses of models A, B, and C obtained using a PN sequence.

From the channel impulse response (see Equation (2)), we can define the root mean square (rms) delay spread ($\tau_{rms}$) as the standard deviation value of the delay reflections of the transmitted signal, weighted proportionally to the energy in the reflection waves. Table 2 shows the rms delay spread expected for each of the three channel models studied in this paper. Figure 10 depicts the channel impulse response of each of three models, computed by means of a PN sequence as explained in Equations (1) and (2). Model A has two paths and models B and C have four paths. Despite having different numbers of paths, models A and C showed a similar delay spread (see Table 2), as the paths were close together in channel C.

Once the channel impulse response is computed, we can derive the channel frequency transfer function as in Equation (10). A key parameter of the frequency characterisation of the channel is the coherence bandwidth. The coherence bandwidth is a statistical measure of the range of frequencies over which the channel can be considered flat (i.e., the bandwidth for which the auto co-variance of the signal amplitude at two extreme frequencies reduces from 1 to 0.5). The coherence bandwidth is inversely proportional to the rms delay spread of the channel (i.e., $B_{c}\approx 1/\tau_{[}rms]$) [20]; but there is no precise relationship between both. However, a widely accepted definition considers the bandwidth over which the frequency correlation is above 0.9; or a more relaxed one considers a frequency correlation of just 0.5:

(10) $$B_{c}\left(0.9\right)=\frac{1}{50\tau_{rms}},B_{c}\left(0.5\right)=\frac{1}{5\tau_{rms}}.$$

**Figure 11:**
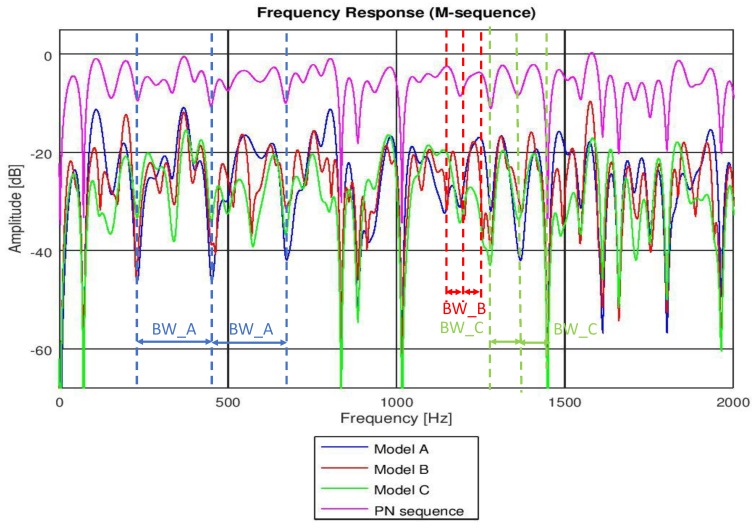
Channel frequency responses of models A, B, and C by means of a PN sequence. An approximate frequency range of the channel coherence bandwidth is shown for each channel model.

In Table 2, we show the coherence bandwidth of each channel model, with frequency correlation above 0.5. In Figure 11, we compare the frequency response of the PN sequence with the frequency response of each channel model computed with the same PN sequence, following Equations (1), (2), and (4). In Figure 11, we do not show the coherence bandwidth (for the sake of practicality), but we underline the bandwidth between consecutive fadings of the channel, which is proportional to the coherence bandwidth for each of the studied models. It is clear that, the higher the rms delay spread of the channel, the narrower the coherence bandwidth and, hence, the higher the probability of acoustic signal distortion.

## 5. Real-Life Acoustic Recording Analysis

In this section, we show the effects of a multi-path static channel on real-life acoustic recorded signals. We, first, underline how the impulse response of a multi-path channel may be estimated by means of an acoustic signal, as if it was a PN sequence. Then, we also show how the frequency selective transfer function of a channel may distort the frequency response of an acoustic signal. Finally, we also show some examples of how the spectral and time distribution of acoustic signals may be changed because of facing different types of multi-path channels.

### 5.1. Propagation on Real-Life Acoustic Recordings

Among all the sample files of recorded ANEs, we have chosen some examples of those with good auto-correlation properties, in order to test whether they are able to estimate the channel response and its impairments, such as delay spread and the resultant distortion in the frequency domain. We also seek ANEs with different spectrum characteristics, in case they have different effects on the multi-path channels.

**Figure 12:**
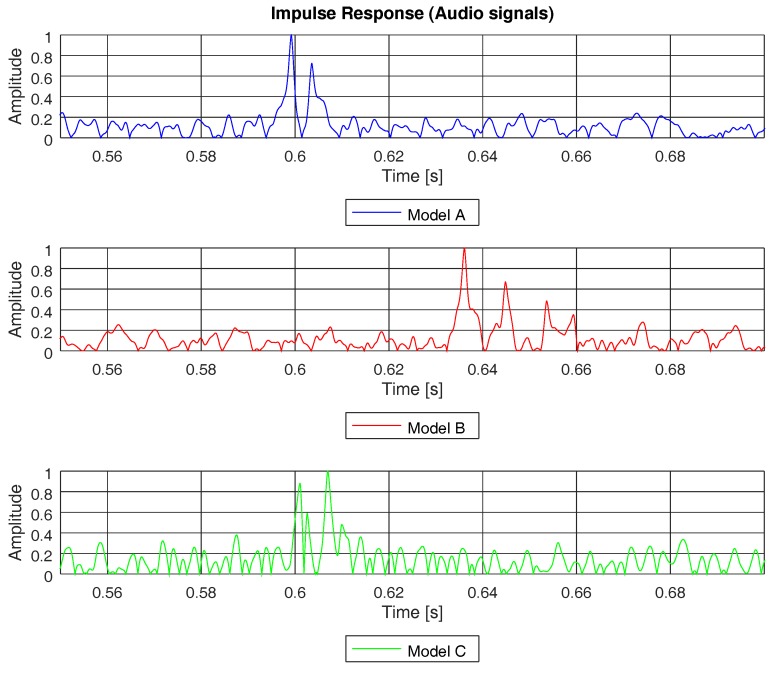
Channel impulse response of models A, B, and C, computed by means of an audio signal of a truck from sensor hb137.

**Figure 13:**
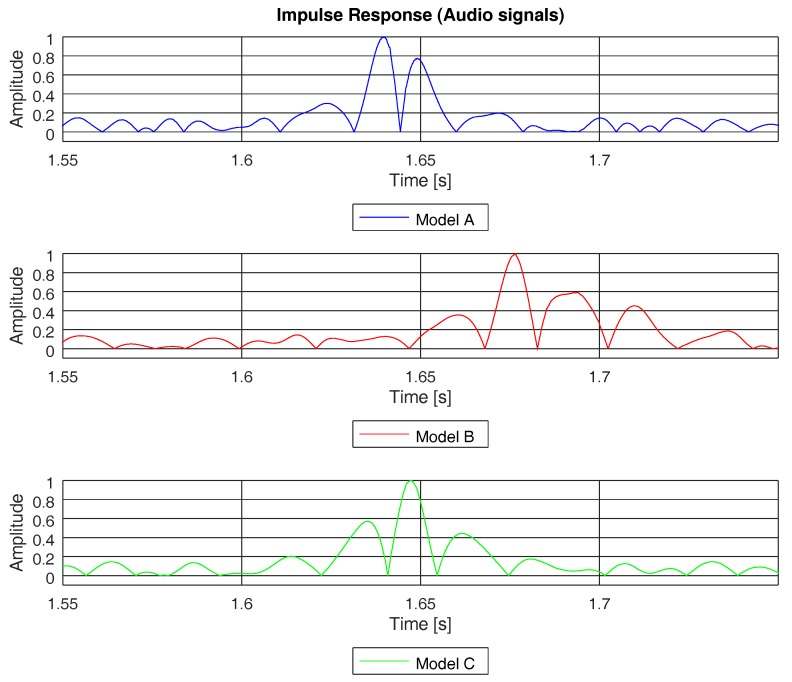
Channel impulse response of models A, B, and C, computed by means of an audio signal of a brake from sensor hb137.

For instance, Figure 12 shows the channel impulse response, computed as in Equation (2), for the three channel models studied in this paper; however, no PN sequence was used in this case. Instead, the same ANE was used to detect the paths of the channel and, therefore, it was able to compute the rms delay spread. In Figure 12, we can see the impulse response of the three channel models computed with an ANE of a truck, with a bandwidth similar to the one shown in the spectrogram of Figure 6d. Due to the wide bandwidth of this ANE and, consequently, the high delay resolution, it was possible to detect the majority of the paths of the three models. This point can be checked if we compare the impulse response in Figure 12 with the channel impulse response computed with the PN sequence (see Figure 10). However, if the bandwidth of the ANE becomes narrow, the delay resolution dismisses and the probability of path detection reduces as well. For instance, Figure 13 depicts the impulse response computed by means of the acoustic signal of a brake, which had a narrow and discontinuous bandwidth, as shown in Figure 6c. Due to the narrow bandwidth and, hence, the poor delay resolution, it was not possible to detect all the paths of channel.

**Figure 14:**
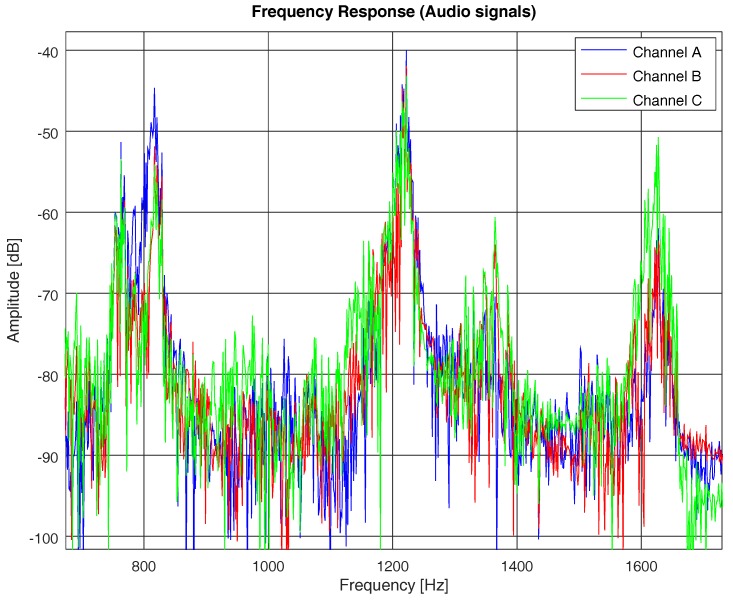
Frequency response of models A, B, and C, computed by means of an audio signal of a siren obtained from sensor hb145.

Regarding the frequency response of the three models, we found that they differed even if they were computed using the same ANE. For instance, Figure 14 plots a portion of the frequency response of the three models computed with an acoustic signal of a siren. It was a signal composed of a number of harmonics, as the spectrogram of Figure 6b shows. In Figure 14, we show three of its harmonics in the range between 800–1600 Hz and we can see that there was a maximum amplitude difference between channel frequency responses of 10 dB. This behavior is not consistent throughout the frequency axis and implies that a model does not always attenuate a frequency component with the same factor, since it computes the frequency response as a linear combination of attenuated paths with a phase that depends on the distance travelled and the frequency (see Equations (9)–(10)).

**Figure 15:**
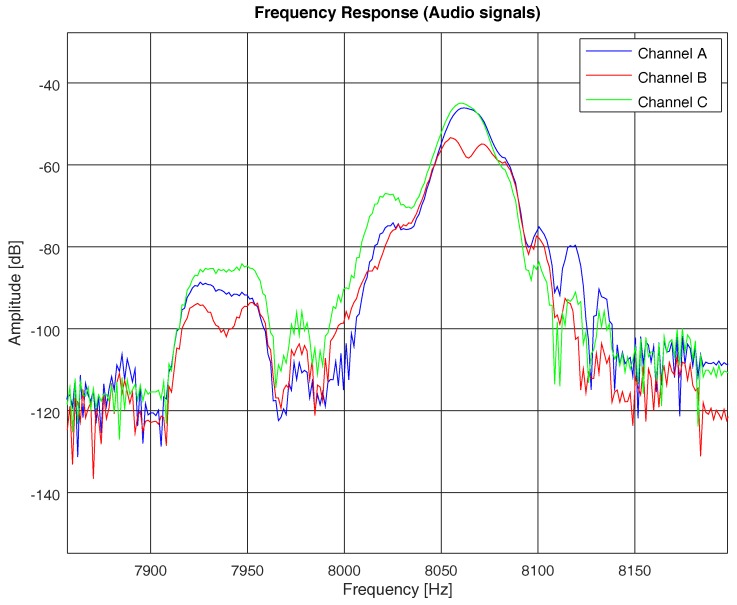
Frequency response of models A, B, and C, computed by means of an audio signal of a brake from sensor hb137.

In addition to the different attenuation of frequency components in each model, we also observed a distortion phenomena which differed between models. For example, Figure 15 depicts the frequency component of an acoustic signal of a brake, for each of the three models, centered at 8 kHz. We can see that, while those models with similar delay spread (i.e., models A and C; see Table 2) had comparable frequency responses, model B had a higher delay spread, which distorted the frequency response. As explained above (see Section 4.3), the higher the delay spread, the narrower the coherence bandwidth of the channel and the higher the probability of signal distortion. The audio signal of a brake, as depicted in Figure 15, had a frequency bandwidth which happened to be flat for models A and C (wide coherence bandwidth), but not flat for model B (narrow coherence bandwidth). Therefore, the brake signal saw models A and C as non-frequency-selective channels, but model B as a frequency-selective channel.

### 5.2. Spectral Distributions over Propagation Channels

**Figure 16:**
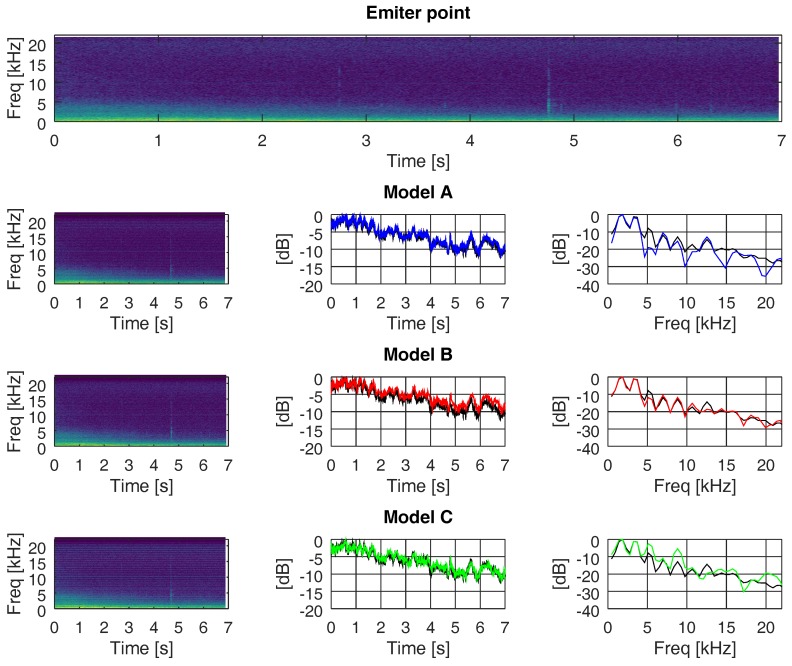
Accumulated energy along time and frequency, through channels A, B, and C, of an audio signal of an aeroplane at sensor hb137.

**Figure 17:**
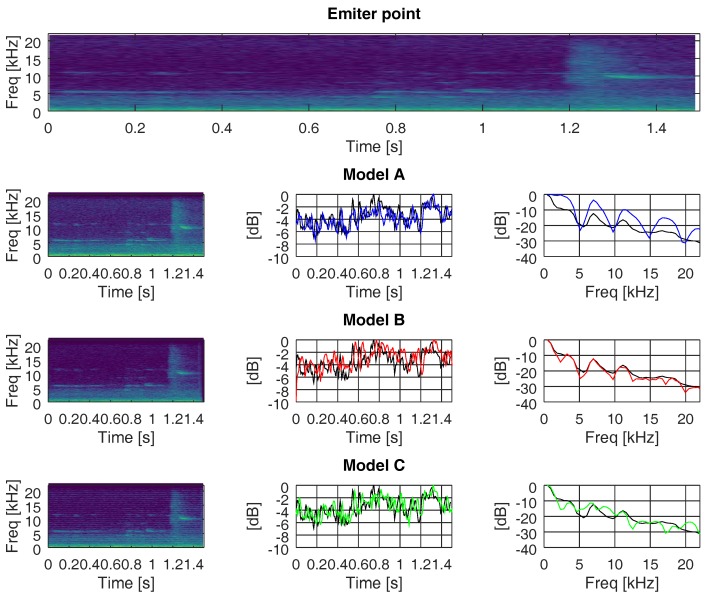
Accumulated energy along time and frequency, through channels A, B, and C, of an audio signal of an brake at sensor hb137.

**Figure 18:**
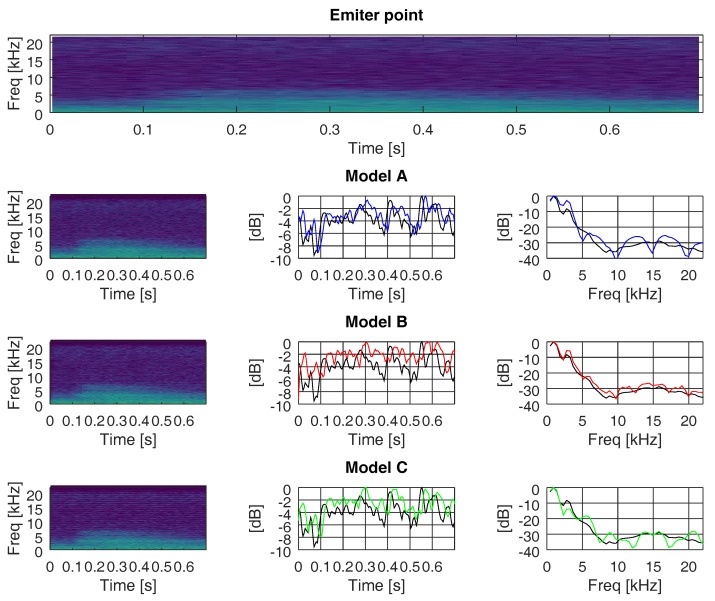
Accumulated energy along time and frequency, through channels A, B, and C, of an audio signal of an truck at sensor hb145.

In this section, we evaluate the changes suffered in the frequency and time domain when the recorded ANEs were propagated through the three different multi-path channels (models A, B, and C, as explained in Section 4.2). Figure 16, Figure 17 and Figure 18 show the outcomes related to different ANEs (i.e., the noise of an aeroplane, a brake, and a truck). For the sake of brevity we only show these three examples, as they are representative of the phenomena we want to outline. For each of them, we show the spectrogram of the emitted signal in the upper plot and then we divide the remaining plot into three rows, one for each model. In the first column, from row two to four, we show the spectrogram of the received signal through models A, B, and C, respectively.

In the second column, from row two to four, we show the accumulated energy throughout the whole bandwidth for each time point, for each propagating model. Finally, in the third column, from row two to four, we show the accumulated energy throughout the whole time duration of the ANE for each frequency, for each propagating model. The spectrogram divides the signal into 40 ms-length segments, which were windowed with a Hanning window to reduce leakage and transformed into the frequency domain by means of a 2048-points Fast Fourier Transform (FFT), diplayed in logarithmic scale. Consecutive segments were overlapped by a factor of 87.5% to maximise the probability of detection. The plots of accumulated energy, in time and frequency, were normalised to the maximum energy and displayed in logarithmic scale. In these plots, we show the accumulated energy of the ANEs prior to being transmitted in black and the accumulated energy after being transmitted through models A, B, and C in blue, red, and green, respectively.

In all three figures, we can observe that the accumulated energy throughout the whole frequency band (middle column) at the emitter was more similar to the accumulated energy at the receiver when propagating through a low delay spread channel (i.e., models A and C), rather than through a higher delay spread channel (i.e., model B). The important factor that enables the spreading of energy over time is not the number of paths but the time distance between them, as well as the weight of each of them.

In all three figures, we can also underline the fact that the accumulated energy throughout the whole time span of the recorded ANEs (right column) approached the original level (i.e., prior to being propagated through the channel) when propagating through a high delay spread channel. In other words, it appears that channels with narrow coherence bandwidths managed to confine the energy into a similar range of values as the original signal. In a different manner, when facing wider coherence bandwidth channels (e.g., models A and C) the accumulated energy in the time domain tended to differ from the original one.

The following step of this analysis will be to systematically analyse all ANEs from both sensors when transmitted through different types of channels (e.g., narrow streets, streets with tall buildings, wide streets with high background noise, and so on). The goal will be to find a systematic repetition of received signal characteristics related to environment factors. Another interesting point will be to study time-variant channels to check whether the time variation factor affects the received signal characteristics.

## 6. Conclusions

The work presented in this paper is a preliminary study, designed to determine the spectro-temporal variations of acoustic signals in the presence of different types of propagation channels in an urban environment. It is intended to be the first detailed analysis applied to raw acoustic data of Wireless Acoustic Sensor Networks, with the aim of reaching a generalisation stage in the near future. The view of the channel over the spectrum of a raw acoustic signal in a real node of a WASN states the influence over the acoustic signal and the changes that apply.

In order to conduct the analysis, several raw acoustic signal, recorded in the framework of the DYNAMAP project, were used; the authors selected pieces of audio coming from sensors surrounded by parks, in order to have an environment similar to open air.

Throughout the research presented in this paper, we have found clear evidence that channel models with different coherence bandwidths affect the frequency response of acoustic signals in different ways. We have shown that, the higher the delay spread of the channel, the narrower the coherence bandwidth and the higher the distortion suffered by acoustic signals. Moreover, the accumulated energy along the frequency axis is more similar to the original signal when transmitted through wide coherence bandwidth channels, rather than through narrow coherence bandwidth channels. However, when computing the accumulated energy along the time axis, those channels with wide coherence bandwidths show more differences from the original signal than those channels with narrow coherence bandwidths.

On one hand, the qualitative evaluations developed in this work present substantial variations, both in terms of spectral distribution energy and in temporal variations due to delay, with a clear influence on the coherence bandwidth. These clear and severe variations in the spectro-temporal description can have severe effects on the detection of anomalous events using the ANED algorithm, as the initial hypothesis of this work stated. On the other hand, the effect of spectral-temporal noise variations on people living in these environments should also be taken into account; do these variations make the noises more annoying? Does perception change when the coefficients of spectral and temporal energy distribution are modified?

## 7. Future Work

The future lines of this work are to focus on the quantification of spectro-temporal variations, depending on the type of channel being used. Research into the best metrics will be pursued, in order to state whether the majority of the anomalous noise events, and even road traffic noise, is modified by the coherence bandwidth, which depends on the multi-path channel described. At the same time, the next natural step of this study will be an analysis focused on the generalisation of study of the channel performance to all available ANEs, where the impact on behavior will be determined as ANED accuracy for different types of channel. Finally, the degree of generalisation of the detection of acoustic events in time-varying propagation environments (with varying numbers and lengths of paths) will be studied, based on the sample of a narrow street with tall buildings or a wider street with more traffic noise.

## Figures and Tables

**Table 1:** List of the nodes of theWireless Acoustic Sensor Network (WASN) deployed in district nine in Milan.

Sensor ID	Street	GPS Coordinates
hb106	Via Litta Modignani	(45.5227587,9.1596847)
hb108	Via Piero e Alberto Pirelli	(45.5144707,9.2107111)
hb109	Viale Stelvio	(45.4929125,9.1919035)
hb114	Via Melchiorre Gioia	(45.4815058,9.1913241)
hb115	Via Fara	(45.4855843,9.1991161)
hb116	Via Moncalieri	(45.5098883,9.1968012)
hb117	Viale Fermi	(45.5089072,9.1802412)
hb120	Via Baldinucci	(45.5032677,9.1686595)
hb121	Via Piero e Alberto Pirelli	(45.5185641,9.2129266)
hb123	Via Galvani	(45.4857107,9.2005241)
hb124	Via Grivola	(45.5179185,9.1943259)
hb125	Via Abba	(45.5028072,9.179285)
hb127	Via Quadrio	(45.4839506,9.1845167)
hb129	Via Crespi	(45.4989476,9.1860456)
hb133	Via Maffucci	(45.4992223,9.1717236)
hb135	via Lambruschini	(45.5024486,9.1548883)
hb136	Via Comasina	(45.5247882,9.1655266)
hb137	via Maestri del Lavoro	(45.518893,9.1997167)
hb138	Via Novaro	(45.5187445,9.1678656)
hb139	Via Bruni	(45.5015796,9.1745067)
hb140	Viale Jenner	(45.4970863,9.1777414)
hb144	Via D’Intignano	(45.5082648,9.2027579)
hb145	Via Fratelli Grimm	(45.5184213,9.2062962)
hb151	Via Veglia	(45.4970074,9.1934109)

**Table 2:** Length and delay of each path, rms delay spread, and coherence bandwidth of each channel model.

	Model A		Model B		Model C	
	Length [m]	Delay [ms]	Length [m]	Delay [ms]	Length [m]	Delay [ms]
**Path 1**	5.0	14.57	8.0	23.32	5.5	16.03
**Path 2**	6.5	18.95	11.0	32.07	6.0	17.49
**Path 3**	-	-	14.0	40.81	7.5	21.86
**Path 4**	-	-	16	46.64	8.5	24.78
$\tau_{rms}$	17.97 ms		34.50 ms		19.74 ms	
$B_{c}\left(0.5\right)$	11.30 Hz		5.79 Hz		10.13 Hz	

## Abbreviations

The following abbreviations are used in this manuscript:

ANE	Anomalous Noise Event
ANED	Anomalous Noise Event Detection
DYNAMAP	Dynamic Noise Mapping
END	European Noise Directive
FIR	Finite Impulse Response
MIMO	Multiple Input Multiple Output
PN	Pseudo-Noise
RRCOSFIR	Root Raised Cosine FIR
RTN	Road Traffic Noise
SNR	Signal-to-Noise Ratio
WASN	Wireless Acoustic Sensor Network
